# Pseudo-document simulation for comparing LDA, GSDMM and GPM topic models on short and sparse text using Twitter data

**DOI:** 10.1007/s00180-022-01246-z

**Published:** 2022-07-09

**Authors:** Christoph Weisser, Christoph Gerloff, Anton Thielmann, Andre Python, Arik Reuter, Thomas Kneib, Benjamin Säfken

**Affiliations:** 1 Georg-August-Universität Göttingen, Göttingen, Germany; 2 Campus-Institut Data Science (CIDAS), Göttingen, Germany; 3 Zhejiang University, Hangzhou, People’s Republic of China; 4 Clausthal University of Technology, Clausthal-Zellerfeld, Germany

**Keywords:** Topic models, Collapsed Gibbs sampler algorithm for the Dirichlet multinomial model, Gamma-Poisson mixture topic model, Latent Dirichlet allocation, Model evaluation, Pseudo-document simulation, Covid-19, Social media, Twitter

## Abstract

Topic models are a useful and popular method to find latent topics of documents. However, the short and sparse texts in social media micro-blogs such as Twitter are challenging for the most commonly used Latent Dirichlet Allocation (LDA) topic model. We compare the performance of the standard LDA topic model with the Gibbs Sampler Dirichlet Multinomial Model (GSDMM) and the Gamma Poisson Mixture Model (GPM), which are specifically designed for sparse data. To compare the performance of the three models, we propose the simulation of pseudo-documents as a novel evaluation method. In a case study with short and sparse text, the models are evaluated on tweets filtered by keywords relating to the Covid-19 pandemic. We find that standard coherence scores that are often used for the evaluation of topic models perform poorly as an evaluation metric. The results of our simulation-based approach suggest that the GSDMM and GPM topic models may generate better topics than the standard LDA model.

## Introduction

Topic models are widely used to extract latent topics in texts, but the most regularly applied models are not well tuned for sparse documents. However, with the rising importance of social media platforms such as Twitter, extracting latent topics from short and sparse texts has become increasingly relevant. Tweets are relatively short, which creates challenges when using standard topic models relying on the inherent assumption that texts are composed as mixtures of latent topics (Mazarura and De Waal 2016).

We compare the performance of the most widely used Latent Dirichlet Allocation (LDA) topic model with the Gibbs Sampler Dirichlet Multinomial Model (GSDMM) and the Gamma Poisson Mixture Model (GPM), which are specifically designed for sparse data and hence presumably more suitable for Twitter data than the LDA with the Pseudo-Document Simulation method.

For evaluating and comparing topic models, standard approaches such as the likelihood-based perplexity metric, coherence scores and top words are insufficient. Chang et al. (2009) shows that the perplexity metric is negatively correlated with measures that are based on human evaluation. Lau et al. (2014) propose coherence scores for automatic topic model evaluation and show that they correlate with the human evaluation of topics. Coherence scores have been widely used for the evaluation and comparison of topic models. In a recent publication, however, Hoyle et al. (2021) provided a detailed critique of coherence scores, showing that high coherence scores do not necessarily correspond to people’s ratings of topic quality. The interpretation by top words, which are the words with a high probability in a topic is an alternative approach to the automatic topic model evaluation. However, this approach relies on subjective human interpretation and is costly and time intensive. We provide a detailed discussion of the shortcomings of the coherence scores and the evaluation by top words in Sect. 2.5.3.

We propose the simulation of pseudo-documents as a new evaluation method to compare LDA, GSDMM and GPM and contrast the results of our method with standard evaluation approaches.

The LDA is a generative process, which assumes that each document in a corpus is generated by a mixture of topics (Blei et al. 2001). Mazarura and De Waal (2016) shows that the LDA model may not perform well when handling short and sparse text data, such as tweets, since these are often just concerned with one specific topic, therefore affecting the validity of the LDA’s main assumption (Alvarez-Melis and Saveski 2016). A remedy for this problem is the pooling of documents in order to create longer pseudo-documents (Mehrotra et al. 2013). Pooling can be done by a feature that all documents share. For tweets, Kant et al. (2020) provides a pooling implementation that uses the hashtags of tweets. However, a limitation of the pooling of tweets is that the obtained topics are estimated for pooled tweets rather than the original tweets.

Two recent alternative generative probabilistic models to the LDA model for sparse text are presented and compared with the LDA model with our proposed Pseudo-Document Simulation method: GSDMM and the GPM model (Yin and Wang 2014; Mazarura et al. 2020). Non-negative Matrix Factorization (NMF) (Févotte and Idier 2011; Pedregosa et al. 2011) is an alternative topic modelling approach that performs well on short and sparse text such as Twitter data (Luber et al. 2021, 2021). In the NMF the estimation of topics is matrix factorization problem with the side constrain of non-negative coefficients, such that no generative probabilistic model is assumed as in the LDA, GSDMM and GPM models.

The GSDMM model is developed by Yin and Wang (2014). Mazarura and De Waal (2016) show that the GSDMM model tends to outperform the LDA model on short and sparse text, when using coherence scores as an evaluation metric. The GPM model is proposed by Mazarura et al. (2020) and compared to the previously developed GSDMM model. Mazarura et al. (2020) also focus on coherence scores to compare the GPM model with the GSDMM model and find that the GPM generates topics with higher average coherence scores. These findings suggest that for short and sparse text the GSDMM model tends to outperform the LDA model, while the GPM model performs better than the GSDMM model. However, because of the shortcoming of coherence scores (Hoyle et al. 2021) these model comparisons are arguably not reliable. To better evaluate the performance of models on sparse documents, we propose the Pseudo-Document Simulation method.

As a case study, we use Covid-19 related tweets. The Covid-19 pandemic makes the analysis of micro-blogs particularly interesting. Twitter data can provide an indicator in real time of how individuals discuss the virus and how policies designed to fight the spread of the virus are perceived (Kant et al. forthcoming; Luber et al. 2021).

The remainder of the paper is structured as follows: Sect. 2 briefly introduces and compares the LDA, GSDMM and GPM topic models, introduces the Covid-19 Twitter data and describes the Pseudo-Document Simulation method for topic model evaluation. Conventional topic model evaluation metrics, namely the evaluation with coherence scores and the evaluation by top words are briefly discussed. In Sect. 3, the outputs of the topic models are compared with the presented evaluation methods. Section 4 provides a short conclusion. Graphs and tables are provided in the appendix. Further details on the hyper-parameters tuning can be found in the supporting materials.

## Pseudo-document simulation for topic model evaluation

This section describes the different topic modelling approaches, the Pseudo-Document Simulation method and conventional topic model evaluation metrics. As a reference for our discussion, an overview of the relevant quantities, notations and assumptions can be found in Table 1.

**Table 1:** Variable list for the LDA, GSDMM and GPM

*K*	Number of topics/clusters
*V*	Number of words in the vocabulary
*D*	Number of documents in a corpus
*d*	Document
$C = \{d_1,\ldots ,d_D\}$	Corpus
$\varvec{\beta }_{k} \sim \text {Dir}(\varvec{\lambda _\beta })$	Word distribution for topic *k*
$\varvec{\theta }_d \sim \text {Dir}(\varvec{\lambda }_\alpha )$	Topic distribution for document *d*
$\varvec{\theta }$	Document topic matrix with rows ${\varvec{\theta }}_{1},\ldots ,{\varvec{\theta }}_{D}$
$\varvec{\lambda }_\beta$	Parameter of the Dirichlet prior on the per-topic word distribution
$\varvec{\lambda }_\alpha$	Parameter of the Dirichlet prior on the per-document topic distributions
$N_{d}$	Number of words for document *d*
$z_{nd} \sim \text {Multinomial}(\varvec{\theta }_d)$	Topic for the *n*th word in document *d*
$z_{d} \sim \text {Multinomial}(\varvec{\theta }_c)$	Topic for the complete document *d*
${\varvec{z}}$	Global topic assignments
$w_{nd}\sim \text {Multinomial}(\varvec{\beta }_{z_{nd}})$	*n*th word in document *d*
$h_{vd}$	Absolute frequency of word *v* in document *d*
$\varvec{\theta }_c \sim \text {Dir}(\varvec{\lambda }_\alpha )$	Distribution over topics for the corpus
$\varvec{\beta }_{z_{nd}}$	Prevalence of the *n*th word for topic *z*
$\eta _{kn} \sim \text {Gamma}(\alpha _{k},\beta _{k})$	Expected frequency of words for all words in topic *k*
$N_{s}$	Number of words in a simulated document
S	Total number of simulated documents
$\varvec{\theta }_{T}$	Theoretical document topic matrix consisting of 1’s and 0’s
$\varvec{\theta }_{S}$	Resulting topic distribution for the pseudo-documents

### Latent Dirichlet allocation

Latent Dirichlet Allocation (LDA) is the standard model for detecting latent topics in documents, implemented, for example, in the Python-package gensim (Řehůřek and Sojka 2010). The LDA model was developed by Blei et al. (2001) as a generative process, which assumes that each document is generated as a mixture of underlying topics, where the continuous-valued mixture proportions are distributed as a latent Dirichlet random variable. A topic is then defined by a distribution over all words in the corpus. In order to avoid double indexing of documents each document is associated with a number counting from 1 to *D*. LDA assumes that each document *d* in a corpus consisting of $d=1,\ldots ,D$ documents is generated as follows:

1. Determine *K* topic distributions as $\varvec{\beta }_{k} \sim \text {Dir}(\varvec{\lambda }_{{\beta }})$ where ${\varvec{\lambda }_\beta }=({\lambda }_{\beta 1},\ldots , {\lambda }_\beta {{V}})$ represents the word relevances in a topic *k*.
2. Determine the distribution over topics for document *d* as $\varvec{\theta }_d \sim \text {Dir}(\varvec{\lambda }_{\alpha })$ where ${\varvec{\lambda }_{\alpha }}=({\lambda }_{\alpha _1},\ldots , {\lambda }_{\alpha _K})$ represents the vector of topic relevances for the corpus.
3. To generate the $N_d$ words $w_{nd}$, $n=1,\ldots ,N_d$ for document *d*,
  - choose a topic $z_{nd} \sim \text {Multinomial}(\varvec{\theta }_d)$ and
  - determine the corresponding words $w_{nd}\sim \text {Multinomial}(\varvec{\beta }_{z_{nd}})$ where $\varvec{\beta }_{z}$ is the vector of word occurrence probabilities *p*(*w*|*z*) given topic *z*.

The hyper-parameters of the LDA are the Dirichlet parameters $\varvec{\lambda }_\beta$ and $\varvec{\lambda }_\alpha$. Note that $\varvec{\beta }$ consists of all (topic-specific) word occurrence probabilities $\beta _{kn}$ while $\varvec{\theta }$ contains all (document-specific) topic occurrence probabilities $\theta _{dk}$, that can be interpreted as the probabilities that a document *d* was generated by a topic *k*. Marginalizing over the latent topics, the generating process for the words of a document *d* can be written as

1 $$\begin{aligned} p(w_{nd}|\varvec{\theta }_d,\varvec{\beta }) = \sum _{k=1}^{K} p(w_{nd}|z=k,\varvec{\beta })p(z=k|\varvec{\theta }_d), \end{aligned}$$

indicating that the LDA model is a mixture model, where the word-specific multinomial models $p(w_{nd}|z,\varvec{\beta })$ are the mixture components and the topic probabilities $p(z|\varvec{\theta }_d)$ are the respective mixture weights.

The generating process for a document *d* can be written as product of word probabilities $p(w_{nd}|\varvec{\theta }_d,\varvec{\beta })$ and integration over $\varvec{\theta }_d$:

2 $$\begin{aligned} p(d | \varvec{\lambda }_\alpha ,\varvec{\beta }) = \int p(\varvec{\theta }_d | \varvec{\lambda }_\alpha ) \left(\prod _{n=1}^{N_d} \sum _{k=1}^{K} p(w_{nd}|z=k,\varvec{\beta })p(z=k|\varvec{\theta }_d)\right) d\varvec{\theta }_d. \end{aligned}$$

The posterior distribution of the hidden variables can be estimated with Gibbs sampling or Variational Inference (Blei et al. 2001).

### Collapsed Gibbs sampler Dirichlet multinomial model

The collapsed Gibbs sampler algorithm for the Dirichlet Multinomial Model (GSDMM) is described by Yin and Wang (2014) as a modification of the LDA model, using a Gibbs sampler on the Dirichlet Multinomial Mixture (DMM) model (Nigam et al. 2000). GSDMM assumes that a document *d* in a corpus consisting of $d=1,\ldots ,D$ documents is generated by a mixture model, such that each document is assumed to be generated by one topic instead of multiple topics as in the LDA (Yin and Wang 2014; Mazarura and De Waal 2016). This process can be described as follows:

1. Determine *K* topic distributions as $\varvec{\beta }_{k} \sim \text {Dir}(\varvec{\lambda }_\beta )$ where ${\varvec{\lambda }_\beta }=({\lambda }_\beta 1,\ldots , {\lambda }_\beta {{V}})$ represents the word relevances in a topic *k*.
2. Determine the distribution over topics for the whole corpus as $\varvec{\theta }_c \sim \text {Dir}(\varvec{\lambda }_\alpha )$ where ${\varvec{\lambda }_\alpha }=({\lambda }_\alpha 1,\ldots , {\lambda }_\alpha K)$ represents the vector of topic relevances for the corpus.
3. For document *d* in the corpus with $d=1,\ldots ,D$ documents:
  - choose a topic $z_{d} \sim \text {Multinomial}(\varvec{\theta }_c)$ and
  - determine the corresponding words $w_{nd}\sim \text {Multinomial}(\varvec{\beta }_{z_{{d}}})$ where $\varvec{\beta }_{z}$ is the vector of word occurrence probabilities *p*(*w*|*z*) given topic *z*.

The hyper-parameters of the GSDMM are $\varvec{\lambda }_\beta$, $\varvec{\lambda }_\alpha$ and the number of iterations of the Collapsed Gibbs Sampler Algorithm.

The DMM generates a document *d* by first selecting a mixture component with regards to the mixture weights $p(z|\varvec{\theta }_c)$. From the conditional distribution $p(d|z,\varvec{\beta } )$, the selected mixture components generate the document *d*. The likelihood of document *d* with the sum of the total probability over all mixture components can be characterized by

3 $$\begin{aligned} p(d) = \sum _{k=1}^{K} p(d|z=k,\varvec{\beta } ) p(z=k|\varvec{\theta }_c), \end{aligned}$$

where *K* is the number of mixture components or topics. The probabilities of the words are independent of their position within a document. Through this, the probability of document *d* generated by topic *k* is given by:

4 $$\begin{aligned} p(d|{z=k},\varvec{\beta } ) = \prod _{w \in d} p(w|{z=k},\varvec{\beta } ). \end{aligned}$$

Instead of the Expectation Maximization algorithm used normally in the DMM, Yin and Wang (2014) proposes the Collapsed Gibbs Sampling algorithm, forming the Gibbs sampler for the DMM model.

### Gamma-Poisson mixture topic model

The Gamma-Poisson Mixture (GPM) model is a topic model proposed by Mazarura et al. (2020), in which a Poisson distribution is used to describe the number of occurrences of a word in the documents with fixed length, instead of a multinomial distribution as in the GSDMM and LDA models. To be more specific, instead of modelling the distribution of a word $w_{nd}$ at position *n* in document *d*, as a multinomial distribution over the vocabulary, the absolute frequency $h_{vd}$ of word *v* in document *d* is modelled with a Poisson distribution. Most topic models in the literature using the Poisson distribution assume that the documents are generated from a mixture of topics as in the LDA. As the GSDMM, the GPM model is a mixture model, assuming that each document is only generated from a single topic instead of a mixture of topics and is hence especially constructed to deal with short text corpora (Mazarura and De Waal 2016). Similar to the GSDMM, the GPM model utilizes a collapsed Gibbs sampler in order to automatically detect the number of topics within a corpus. The probabilistic generative process for a document *d* is characterized as follows:

1. Determine the expected frequency of words $\eta _{k{v}} \sim \text {Gamma}(\alpha _{k},\beta _{k})$ for all words *v* in topic *k* for $k=1,\ldots ,K$ topics. $\alpha _{k}$ is the shape parameter and $\beta _{k}$ the scale parameter of the respective $\text {gamma}$ distribution for topic *k*.
2. Determine the distribution over topics for the corpus as $\varvec{\theta }_c \sim \text {Dir}(\varvec{\lambda }_\alpha )$, where ${\varvec{\lambda }_\alpha }=({\lambda }_\alpha 1,\ldots , {\lambda }_\alpha K)$ represents the vector of topic relevances for the corpus.
3. For document *d* in the corpus with $d=1,\ldots ,D$ documents:
  - Choose a topic $z_{d} \sim \text {Multinomial}(\varvec{\theta }_c)$ and
  - Determine the corresponding word counts ${h_{vd}}\sim \text {Poisson}(\eta _{k{v}})$ where the rate parameter $\eta _{k{v}}>0$ represents the expected frequency of word *v* in topic *k*.

As in the GSDMM, the DMM generates a document *d* by first selecting a mixture component with regard to the mixture weights $p(z|\varvec{\theta }_c)$. The likelihood of a document is then given similar to the GSDMM model:

5 $$\begin{aligned} p(d) = \sum _{k=1}^{K} p(d|z=k,\varvec{\eta }_k) p(z=k|\varvec{\theta }_c), \end{aligned}$$

where *K* denotes the total number of topics. Note that, both the GSDMM and the GPM use a Naive Bayes framework, in which the word frequencies are considered independent within each topic. The conditional probability of a document, given a certain topic *k* and all topic-specific rate parameters as $\varvec{\eta }_k = (\eta _{k1}, \ldots , \eta _{kV})$, is thus denoted by:

6 $$\begin{aligned} { p(d|z = k, \varvec{\eta }_k) = \prod _{v = 1}^V p(h_{vd}|\eta _{kv}).} \end{aligned}$$

$p(h_{vd}|\eta _{kv})$ stands for the probability of a certain word count $h_{vd}$ in document *d* and $\eta _{kv}$ denotes the expected frequency of word *v* in topic *k* as the rate parameter of a Poisson distribution. Similar to the GSDMM model, a collapsed Gibbs sampler is incorporated in the GPM model in order to learn the latent topics hidden in the documents.

The main difference between the GSDMM and the GPM is that the word frequencies $w_{nd}$ in the GPM model are modeled with independent Poisson distributions, while the word frequencies in the GSDMM model are modeled jointly with a multinomial distribution. As a result, the GPM model assumes a Gamma prior distribution as a conjugate prior for the Poisson distribution, while GSDMM uses as Dirichlet distribution as a conjugate prior for the multinomial distribution.^1^

#### Online variational Bayes algorithm for LDA

In general, Variational Bayesian methods are based on the idea of approximating posterior densities by optimization over a previously posited family of distributions. The online Variational Bayes algorithm for LDA was proposed by Hoffman et al. (2010) and is based on a mean-field approach where the posterior density is approximated by $q(\varvec{\beta }, \varvec{\theta }, {\varvec{z}})$ in order to maximize the Evidence Lower Bound ${\mathcal {L}}$:

7 $$\begin{aligned}{\mathcal {L}}(C, \varvec{\gamma }, \varvec{\kappa }, \varvec{\psi }) &{\mathop{=}\limits^{\text{def}}}{\mathbb {E}}_q[\log p(C, \varvec{\theta }, \varvec{\beta }, {\varvec{z}} | \varvec{\lambda }_{{\alpha }}, \varvec{\lambda }_{{\beta }})] - {\mathbb {E}}_q[\log q(\varvec{\beta }, \varvec{\theta }, {\varvec{z}})] \\ &\quad \le \log p(C| \varvec{\lambda }_{{\alpha }}, \varvec{\lambda }_{{\beta }}) \end{aligned}$$

More specifically, variational parameters $\varvec{\gamma }$, $\varvec{\kappa }$ and $\varvec{\psi }$ are introduced such that $q(\varvec{\beta }, \varvec{\theta }, {\varvec{z}}) = \prod _{k=1}^K q(\varvec{\beta }_k) \prod _{d = 1}^D q(\varvec{\theta }_d)q({\varvec{z}}_d)$ where $q(\varvec{\beta }_k) = \text {Dir}(\varvec{\beta }_k; \varvec{\gamma }_k)$ and $q(\varvec{\theta }_d) = \text {Dir}(\varvec{\theta }_d; \varvec{\kappa }_d)$ and $q({\varvec{z}}_d) = \prod _{n = 1}^{N_d} \text {Multinomial}({\varvec{z}}_{n, d}; \varvec{\psi }_{n, d})$. Subsequently, ${\mathcal {L}}$ can be optimized using coordinate ascent for the variational parameters (Blei et al. 2016). However, the authors of Hoffman et al. (2010) propose to use an online variational inference algorithm similar to classic coordinate ascent, which can be interpreted as stochastic optimization with natural gradient updates for the parameter $\varvec{\gamma }$. The proposed algorithm is included in the appendix. The point estimates used for the implementation of this algorithm are the expectations of the distributions of the respective variational parameters.

### Collapsed Gibbs sampler

The collapsed Gibbs sampler, introduced by Liu (1994) for Bayesian missing data problems, is a version of the Gibbs sampler that uses full conditional distributions of only a subset of all variables within a model to draw the components from the generated samples. To obtain those collapsed conditional distributions, one can marginalize over selected parameters and hence integrate out variables that are not of direct interest. Thereby sampling can be facilitated or the number of sampling steps in each iteration of the Gibbs sampler reduced, which possibly yields computational advantages (Table 2).

**Table 2:** Variable list for the collapsed Gibbs sampler

$N_{d}$	Number of words for document *d*
${\varvec{N}}^{k}$	Total number of words in documents assigned to topic *k*
${\varvec{N}}_w^{k}$	Number of times word *w* appears for topic *k*
${{\varvec{N}}}_k = ({\varvec{N}}_w^k)_{w=1}^{V}$	Vector comprising all the counts of specific words for topic *k*
${\varvec{H}}_w^d$	Number of times word *w* appears in document *d*
${\varvec{M}}^{k}$	Total number of documents assigned to topic *k*
${{\varvec{M}}} = ({\varvec{M}}^k)_{k=1}^{K}$	Vector comprising total number of documents for each topic
Additional index $-d$	Document *d* is ignored for the corresponding count
$\lambda _\alpha$	Component of the symmetric vector $\varvec{\lambda _\alpha } = (\lambda _\alpha , \ldots , \lambda _\alpha )$
$\lambda _\beta$	Component of the symmetric vector $\varvec{\lambda _\beta } = (\lambda _\beta , \ldots , \lambda _\beta )$
$\alpha$ and $\beta$	Uniformly fixed parameters of the Gamma priors in GPM

Capitalized bold letters indicate counts

#### Collapsed Gibbs sampler for GSDMM

The goal of inference is to determine the posterior distribution $p(\varvec{\beta }, \varvec{\theta }_c, {\varvec{z}} | C, \varvec{\lambda }_\alpha , \varvec{\lambda }_\beta )$ of the latent variables given the entire corpus $C = \{d_1,\ldots ,d_D\}$, and hyper-parameters $\varvec{\lambda }_\alpha$ and $\varvec{\lambda }_\beta$, which we will partially suppress in the notation. Marginalizing over $\varvec{\beta }$ and $\varvec{\theta }_c$ and neglecting various constant factors yields a simple equation for the conditional distribution of the single entries of ${\varvec{z}}$; see Yin and Wang (2014) for details on the derivation. More precisely, under the assumption of symmetric Dirichlet priors, i.e. $\varvec{\lambda }_\alpha = (\lambda _\alpha , \ldots , \lambda _\alpha )$ and $\varvec{\lambda }_\beta = (\lambda _\beta , \ldots , \lambda _\beta )$, one formally obtains the following result for the distribution of the topic $z_d$ given the corpus and all other topics per document ${\varvec{z}}_{-d}$:

8 $$\begin{aligned} p(z_{d} = k| {\varvec{z}}_{-d}, C) \propto \frac{{\varvec{M}}^{k,-d} + \lambda _{\alpha }}{D-1 + K\lambda _{\alpha }} \frac{\prod _{w \in d}\prod _{j=1}^{{\varvec{H}}_w^d} ({\varvec{N}}_w^{k, -d} + \lambda _{\beta } +j -1)}{\prod _{i=1}^{N_d}({\varvec{N}}^{k, -d} + \lambda _\beta + i -1)} \end{aligned}$$

Here ${\varvec{M}}^{k}$ denotes the number of documents assigned to topic *k*, and ${\varvec{N}}^{k}$ is the number of words in topic *k*. Additionally, ${\varvec{N}}_w^{k}$ is the number of occurrences of word *w* in topic *k* and ${\varvec{H}}_w^d$ equals the number of times word *w* appears in *d*. The additional index $-d$ indicates that the specific document *d* is neglected for the corresponding count. Based on this result, the conditional distribution of the components of ${\varvec{z}}$, given by Eq. (8) up to proportionality, can be used to perform Gibbs sampling with significantly reduced computational expense compared to an immediate realization of the Gibbs sampler. The concrete algorithm is included in the appendix 1.

Because a Dirichlet distribution is used as prior for the multinomial distribution in GSDMM, the posteriors for $\varvec{\beta }$ and $\varvec{\theta }_c$ are simply given by $p(\varvec{\beta }_{k} |{\varvec{z}}, C) = {\text {Dir}(\varvec{\beta }_{k}| {{\varvec{N}}}_k + \varvec{\lambda }_\beta )}$ with ${{\varvec{N}}}_k = ({\varvec{N}}_w^k)_{w=1}^{V}$ and $p(\varvec{\theta }_c |{\varvec{z}}, C) = \text {Dir}(\varvec{\theta }_c| {{\varvec{M}}} + \varvec{\lambda }_\alpha )$ where ${{\varvec{M}}} = ({\varvec{M}}^k)_{k=1}^{K}$. Subsequently, the mean of those posterior Dirichlet distributions are commonly used as a point estimate for $\varvec{\beta }$ and $\varvec{\theta }_c$.

Additionally, experimental results Yin and Wang (2014) show that the Gibbs sampling algorithm for the GSDMM can retrieve the true number of topics when initially provided with more topics than actually present, as the fraction of non-empty clusters decreases within several iterations. This finding is supported by the structure of Eq. (8) because the first factor causes documents to be assigned to topics which already have a large quantity of other documents, while the second factor implies that the document is assigned to a topic with similar words. Therefore, the number of iterations directly influences the number, size and heterogeneity of the clusters.

#### Collapsed Gibbs sampler for GPM

As the structure of the GPM is very similar to GSDMM, and because this model also relies on conjugate priors for the hyper-parameters, the conditional distributions for the collapsed Gibbs sampling algorithm in this case can be derived similarly to the previous examples via integrating out the $\eta _{kn}$ and $\varvec{\theta }_c$ (Mazarura et al. 2020). Assuming a symmetric Dirichlet prior $\varvec{\lambda }_\alpha = (\lambda _\alpha ,\ldots ,\lambda _\alpha )$ as previously, and $\alpha _k = \alpha$, and $\beta _k = \beta$ for all *k*, one obtains the following result:

9 $$\begin{aligned} p(z_{d} = k| {\varvec{z}}_{-d}, C)&\propto  {} \frac{{\varvec{M}}^{k, -d} + \lambda _\alpha }{K-1 + K\lambda _\alpha } \frac{\beta ^{N_d}}{{\prod _{w \in d} ({\varvec{H}}_w^d!)}} \frac{({\varvec{M}}^{k, -d}\beta + 1)^{({\varvec{N}}^{k, -d} + V\alpha )}}{({\varvec{M}}^{k, -d}\beta + \beta +1)^{({\varvec{N}}^{k, -d} + N_d+ V\alpha )}} \\ &\quad \prod _{v =1}^{V}\prod _{j=1}^{{\varvec{H}}_d^v} ({\varvec{N}}_v^{k, -d} + \alpha + j -1) \end{aligned}$$

Subsequently, this expression can be used to perform Gibbs sampling for ${\varvec{z}}$. The corresponding algorithm, as proposed by Mazarura et al. (2020), is identical to the Gibbs sampling algorithm for GSDMM except that $z_d$ (“Appendix 1”) is sampled according to Eq. (9). Furthermore, normalization of the word frequencies per document ${\varvec{H}}_w^d$ is proposed by Mazarura et al. (2020) since Poisson distributions may be used to describe the probability to observe a certain number of events within a fixed interval. Therefore, ${\varvec{H}}_w^d$ can be replaced by $\varvec{\tilde{H}}_d^w = \frac{N \cdot {\varvec{H}}_w^d}{\sum _{r=1}^V {\varvec{H}}_r^d}$ where the hyper-parameter *N* is a length fixed uniformly for all documents and $\varvec{\tilde{H}}_d^w$ is rounded off to the nearest integer. Additionally, as for GSDMM, the posterior of $\eta _{kv}$ and $\varvec{\theta }_c$ are immediately accessible and given by $p(\eta _{kv} |{\varvec{z}}, C) = \text {Gamma}({\varvec{N}}_v^k + \alpha , \beta /({\varvec{M}}^k \beta +1))$ and $p(\varvec{\theta }_c |{\varvec{z}}, C) = \text {Dir}(\varvec{\theta }_c| {{\varvec{M}}})$. As previously, we use the posterior means as point estimates.

Moreover, experimental results indicate that the collapsed Gibbs sampling procedure for GPM is even better suited for identification of the true number of topics than GSDMM when provided with an appropriately large number of initial topics (Mazarura et al. 2020). The number of iterations for the Gibbs sampler plays a similar role as for GSDMM.

### Pseudo-document simulation and model evaluation

This section provides a brief description of the text data and pre-processing steps that have been applied in this study. Subsequently, the Pseudo-Document Simulation Method for the topic model evaluation is presented.

#### Data and pre-processing

For our source documents, we stream tweets from the social media platform Twitter. The Tweepy API for Python used in this work collects about 1% of all tweets drawn in real time (Roesslein 2009). The data was streamed daily for 1 week and consists of tweets posted from the 14^th^ to the 21^st^ of July 2020. Only tweets posted from the United States of America are used, excluding Alaska and Hawaii. The filter function of the Tweepy API is used to specifically search for tweets related to the Covid-19 pandemic by choosing keywords (“covid", “corona" and “covid-19") included in the tweets.

In order to get interpretable results the data is pre-processed as follows. First, all tweets that are not written in English are removed using the inherent language detection of the tweepy API. Second, all emojis, hyperrefs and tags are removed. Third, the documents are tokenized, meaning that the sentences are transformed into tokens (individual words), divided by commas. Fourth, all parts in the data which do not contain useful information, so-called stopwords, are removed, using the library of the *spacy*-package. All words, inherently not containing any useful semantic meaning as e.g. “it" are hence removed from the corpus. Additionally, all words that are shorter than two letters are removed. Fifth, the data is lemmatized which reduces all words into their non-conjugated forms (Korenius et al. 2004). We do not apply further data cleaning on the corpus to compare the topic models based on typical Twitter data. In the last pre-processing step, bigrams are implemented. Bigrams are often used in addition to unigrams in order to enhance text classification (Tan et al. 2002; Wang and Manning 2012; Bekkerman and Allan 2004). Bigrams combine words, which are used in tandem for example “United States" or “New York" and transform them into one word. The minimal occurrence threshold for the bigrams was set to 30. Hence unigrams and bigrams with an occurrence of $>30$ are included in the vocabulary.

#### Pseudo-document simulation

We propose a novel method for topic model evaluation by simulating labelled pseudo-documents. For every model, a unique pseudo-document corpus is generated. Initially, the LDA, GSDMM and GPM topic model are fitted on the source data, such that a word-probability matrix $\varvec{\beta }$ is generated for each topic model. The columns of the respective resulting matrices contain the probability distributions over words for each topic ($\varvec{\beta } = (\varvec{\beta }_{1}, \varvec{\beta }_{2},\ldots , \varvec{\beta }_{K})$). Note that each column represents a topic and the rows represent all words in the vocabulary. Hence, $\varvec{\beta }$ consists of all (topic-specific) word occurrence probabilities $\beta _{kn}$. In the following, the probabilities in $\varvec{\beta }$ from each model and a theoretical document topic matrix $\varvec{\theta }_{T}$ are used to simulate short and sparse labelled pseudo-documents that are similar to tweets and thus similar to the source data. Subsequently, the models are fitted on the labelled pseudo-documents and the labels are used to evaluate and compare the performance for each model.

The scraped tweets are used to generate a representative distribution for the number of words in our sample of tweets. Based on the distribution in Fig. 6, we select 4–30 words per simulated tweet. Note that the document length is chosen based on the length of the used pre-processed corpus. The number of words for each simulated document $N_{s}$ is sampled from a discrete uniform distribution for the specified range [4, 30]. Thereby $s = (1, 2,\ldots , S)$ represents the simulated documents, with $S=100,000$ being the total number of documents that are simulated.

$N_{s}$ words are drawn based on the word probability distribution for topic *k* from $\varvec{\beta }_{k}$. The simulation is based on a theoretical $\varvec{\theta }_{T}$ matrix that assigns $\frac{S}{K}$ documents to each topic *k*. Thus, we create labelled documents and simulate a fixed number of 5000 documents per topic. In detail, for one topic model the Pseudo-Document Simulation proceeds as follows:

1. Determine $\varvec{\beta }$ for the sample of source tweets.
2. For topic *k* in topics $k=1,\ldots ,K$:
  - For pseudo-document *s* in pseudo-documents $s=1,\ldots ,S/K$:
    (i) Determine the number of words $N_{s}$ from the range [4,30].
    (ii) Determine $N_{s}$ words $w_{ns}$ from the word probability distribution for topic *k* from $\varvec{\beta }_{k}$

The rows of the theoretical matrix $\varvec{\theta }_{T}$ represent the documents that are created, whereas the columns represent the number of topics. The elements of $\varvec{\theta }_{T}$ are 1 if the document draws words regarding the respective topic and 0 otherwise. Hence, $\theta _{T_{k}}$ is a vector with $\frac{S}{K}$ ones and $S-\frac{S}{K}$ zeros. With this simulation, a pseudo-corpus is created, which contains pseudo-documents for each of the topics. Due to the set up of the simulation, the number of simulated documents is equally distributed over topics. Thereby the simulated documents contain $\frac{S}{K}$ words representative of a specific topic. The order of words within the document is created randomly. Note that the created pseudo-documents are pseudo-tokenized, since the documents are already created with tokenized words. Additionally only the word co-occurrences are important for the extractions of clusters by different topic models. The document topic matrix $\varvec{\theta }_{S}$ is obtained by estimating the models with the simulated data. In the following $\varvec{\theta }_{S}$ will be compared with $\varvec{\theta }_{T}$. The described simulation is implemented for each of the LDA, GSDMM and GPM model.

#### Model evaluation

**Fig. 1:**
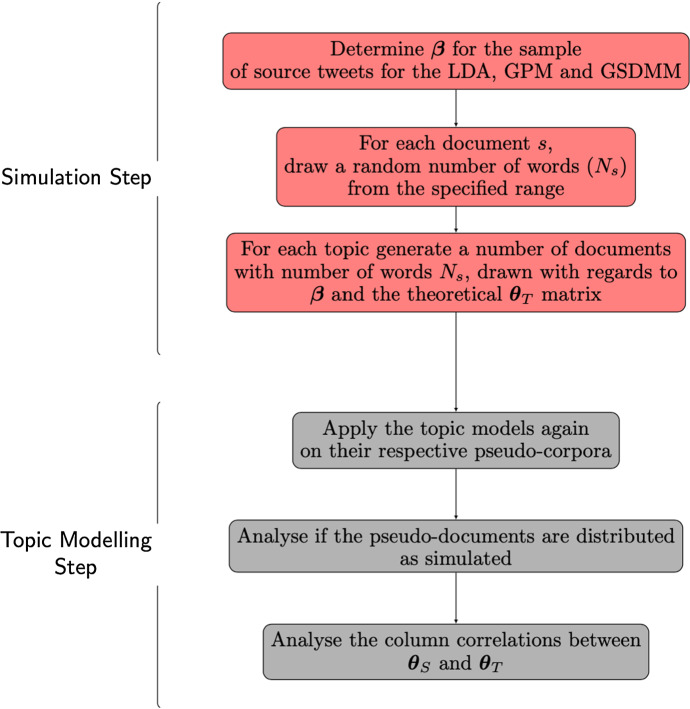
Process of the Document Simulation and Analysis

The topic models are then applied again on the respective simulated pseudo-corpora. A well performing topic model, that is applied on the simulated data, should generate topics each containing about the same number of documents, because for each topic the same number of documents are created. To evaluate how the topic models perform on the simulated data, it is hence firstly evaluated, if the number of documents are equally distributed over topics. While the resulting topics are hopefully closely related to the topics from which the simulated documents are created, direct topic classification of the documents is not possible, as the resulting topics are not necessarily identical to the initial topics from the document simulation. Hence, measures like accuracy or precision cannot be calculated as all three topic modelling approaches do not yield a direct classification. As a remedy, we calculate the column correlations of the $\varvec{\theta }_{S}$ matrix of each model with the theoretical $\varvec{\theta _{T}}$ matrix, which contains the true document topic prevalence values. High correlation values indicate that a simulated topic can be mapped to a topic in $\varvec{\theta _{T}}$. We map the columns of the $\varvec{\theta }_{S}$ matrix to the columns of the theoretical $\varvec{\theta }_{T}$ matrix with the highest correlations in each case. The whole process of simulating labelled pseudo-documents, estimating topics and evaluating the models is visualized below (Fig. 1):

Our proposed method allows us to evaluate and compare the overall performance of the different topic models objectively on concrete data. Therefore, we provide an alternative to the conventional evaluation methods that have various shortcomings and will be discussed in the next section. To compare the models, we estimate the word-probability matrix $\varvec{\beta }$ for each model with the original data, rather than constructing a theoretical $\varvec{\beta }$ matrix that is used by each model. As a result, our method allows us to evaluate the overall model performance. However, we cannot distinguish whether an improved performance comes from a better estimation of $\varvec{\beta }$ or from the ability of the model to retrieve $\varvec{\theta _{T}}$.

### Conventional topic model evaluation metrics

This section discusses conventional evaluation methods. First, the UMass-coherence score, which is a widely used metric for topic model evaluation, despite of its various shortcomings, is briefly described. Second, the labelling of the top words of the topics as a subjective but also common method is discussed.

#### Coherence score

Coherence scores are often used as an automatic scoring metric for evaluating a topic models performance and hence are frequently used for hyper-parameter optimization. Coherence, a systematic or logical consistency, is defined in terms of topics as the co-occurrence of words with similar semantic meaning within the same document. The coherence score hence measures to which extend a topic consists of words with high probabilities in $\varvec{\beta }$ that also occur together with high probability in the data. A topic that assigns high probabilities to words or phrases that do not often occur together in the data^2^ would be scored as a *bad* topic. Rosner et al. (2014) proposes the intrinsic UMass measure, which calculates the topic coherence as co-occurrences of a topics most prevalent words within all documents as follows:

10 $$\begin{aligned} C(z) = \sum _{j=2}^{M} \sum _{i=1}^{j-1} \log \left( \frac{p(w_{j},w_{i}|z) + \epsilon }{p(w_{i}|z)}\right) . \end{aligned}$$

$p(w_{j},w_{i})$ denotes the probability that the words $w_{j}$ and $w_{i}$ co-occur within a document, calculated as the number of documents containing both words divided by the total number of documents. $p(w_{i})$ denotes the probability that word $w_{i}$ occurs in a document and is hence calculated as the number of documents containing word $w_{i}$ divided by the total number of documents. These probabilities are dependent on topic *z*, since the coherence metric is calculated for each topic. *M* denotes the number of words with the highest probabilities in topic *z* that are evaluated. These words are obtained by ordering the probabilities in $\varvec{\beta }$ for the respective topic in descending order. Therefore, when evaluating different topic models, care should be taken to use the same value of *M*, otherwise the results may be biased. Choosing a very small value of *M* of e.g. 2 would thus only take the two most probable words into account and could lead to a topic consisting of two very coherent words but otherwise incoherent words. In line with Rosner et al. (2014), we set *M* to 20 such that topic *z*’s 20 most probable words are taken into account as Röder et al. (2015) found that evaluation of the topic quality is harder, if *M* is small. $\epsilon$ is set to $\frac{1}{M}$ as Stevens et al. (2012) found that smaller values for $\epsilon$ lead to a better performance of the coherence measure and generally ensures that the logarithm of zero is not taken. Coherence scores are a non-subjective measure of the created topics interpretability. However, a model can also generate good coherence scores, while creating completely uninformative topics by evaluating common word co-occurrences of non-informative words, e.g. stopwords. Additionally, good coherence scores can be also generated when a topic model results in multiple, but very similar topics. Furthermore, Röder et al. (2015) provides evidence that the UMass coherence measure can have low correlations with human interpretability of topics when dealing with smaller word sets.

#### Top words

A subjective evaluation method of topic models is the interpretation of top words, i.e. words with the highest probability in a topic and labelling of topics by humans. Chang et al. (2009) tests this evaluation method by asking individuals to detect intruder words. The test can consist of e.g. adding an intruder word as one of the top words in a given topic, or adding an intruder topic as one of the three most relevant topics. An intruder word is a word, which does not belong to the topic associated with the words. An intruder topic is a topic with low relevancy. The results show that individuals tend to show high capability in the detection of intruders. Tables and wordclouds that visualize the top words are provided in “Appendix 1”.

## Empirical evaluation

In the following, the empirical results of the LDA, GSDMM, and GPM models are discussed. The top words for 20 topics are compared and the clusters are manually labeled in order to compare the interpretability of the topics for each model. The coherence scores for different numbers of topics *K* are compared between the models. Lastly, we apply the Pseudo-Document Simulation method and analyze the model performance based on the pseudo-documents.

For the LDA model fixed symmetric priors are used for the hyper-parameters $\varvec{\lambda }_{\beta }$ and $\varvec{\lambda }_\alpha$, such that each element takes the value $\frac{1}{K}$. This is in line with the default specification in the Python-package gensim (Řehůřek and Sojka 2010). For the GPM and GSDMM we use the recommended hyper-parameter specification in Mazarura et al. (2020). We specify the hyper-parameters of the GSDMM model so that the elements in $\varvec{\lambda }_{\beta }$ and $\varvec{\lambda }_\alpha$ are 0.1. For $\alpha _{k}$ and $\beta _{k}$ in the GPM model we use the value 0.001, which is equivalent to setting non-informative priors in a Bayesian context (Mazarura et al. 2020). In addition, we use 0.1 for the elements in $\varvec{\lambda }_\alpha$.

**Fig. 2:**
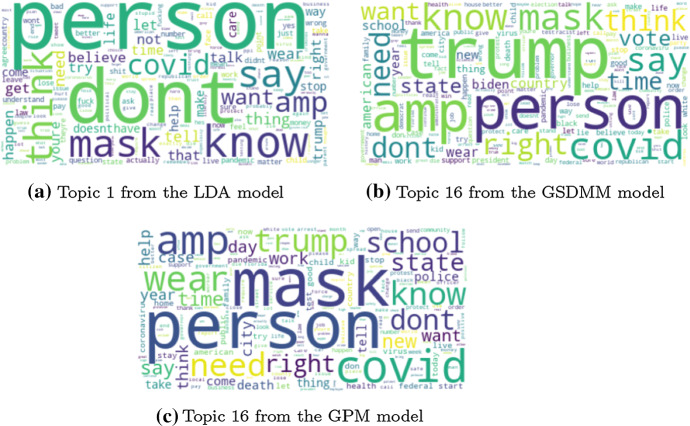
Wordclouds for the topics most related to Covid-19

**Fig. 6:**
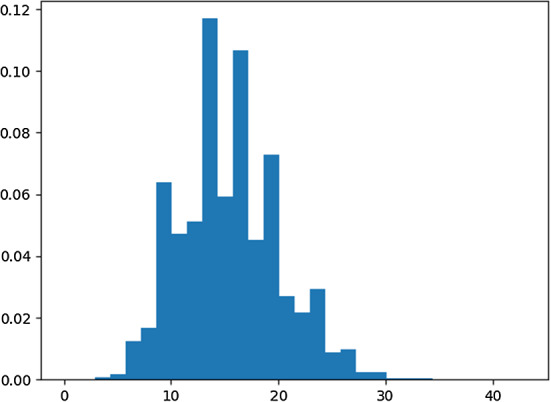
Number of words in the documents

The 10 top words for each of the three models over 20 topics are visualized in Tables 3, 4 and 5 in “Appendix 1”. We manually labeled each of the topics based on the top words. Note that the horizontal lines in the tables indicate that we cannot clearly assign a label to the respective topic based on the displayed words. When analyzing the top words of the models, we observe that the LDA model seems to produce topics containing many irrelevant and unrelated words. This makes the topic labelling process challenging. The GSDMM and the GPM model produce topics that can be more easily interpreted and manually labeled. Figure 2 shows that the Covid topics generated by GSDMM and the GPM appear to contain more informative words than the LDA model. These results contrast with those based on a comparison of the coherence scores, which suggests a performance advantage of the LDA model over the GSDMM and GPM models.

Figure 9 shows that the LDA model seems to perform better than the GSDMM and the GPM model based on different average coherence values, for a different number of topics. Larger coherence scores are supposed to indicate a better model performance and thus the LDA seems to outperform the other models for any number of topics. Therefore, the comparison of the coherence scores seems to suggest that the LDA model outperforms both, the GSDMM and the GPM model, despite that the two latter models are specifically designed to handle sparse data corpora like the tweets used in this work.

To avoid the problems associated with the use of coherence scores and the issues of manually evaluating the models results, we suggest a method that compares the model performance based on pseudo-documents. The documents for the pseudo-corpora are simulated as described in Sect. 2.5 and the number of initial topics is set to 20. We generated 100,000 documents in total for 20 topics and thus 5000 documents per topic. Therefore, when the topics are initially created, the simulated documents are by design distributed uniformly across the topics. Because of this design, a perfect topic model that is estimated on the simulated document would result in an evenly distributed document assignment, with each topic being discussed in 5000 documents. For the evaluation of the results of the topic models, we use the document topic prevalence scores. Each simulated document is labeled as “belonging” to a topic if the prevalence of the document for that topic is greater than the prevalence of that document for any other topic.

**Fig. 7:**
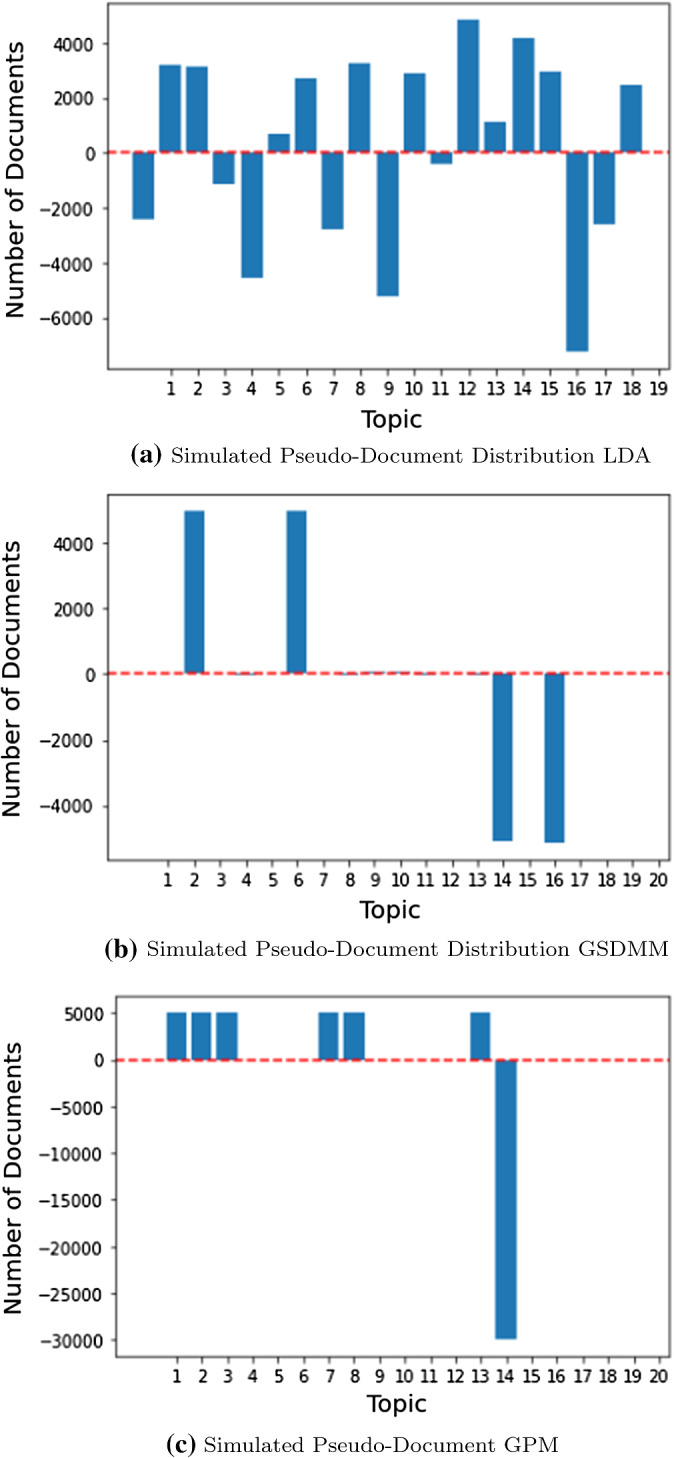
Deviation from the 5000 documents to which each topic should be assigned

The deviation from the 5000 documents that a perfect model should find is represented in Fig. 7. The simulated documents for LDA models shows the highest deviation from a uniform distribution of the pseudo-documents. In addition, for the LDA model over 20,000 documents are not assigned to any topic because a prevalence score of 0.05 is estimated for every topic. Hence, there does not exist a maximum which could be used for determining a hard assignment so that these 20,000 documents cannot be allocated. Despite the LDA model outperforming the other two models based on coherence scores, these first results already confirm what can be seen in the manual analysis. The GSDMM and the GPM models seem to perform better, since a large proportion of topics contain 5000 documents. The GPM model also seems to result in a more uniformly distributed document assignment.

**Fig. 8:**
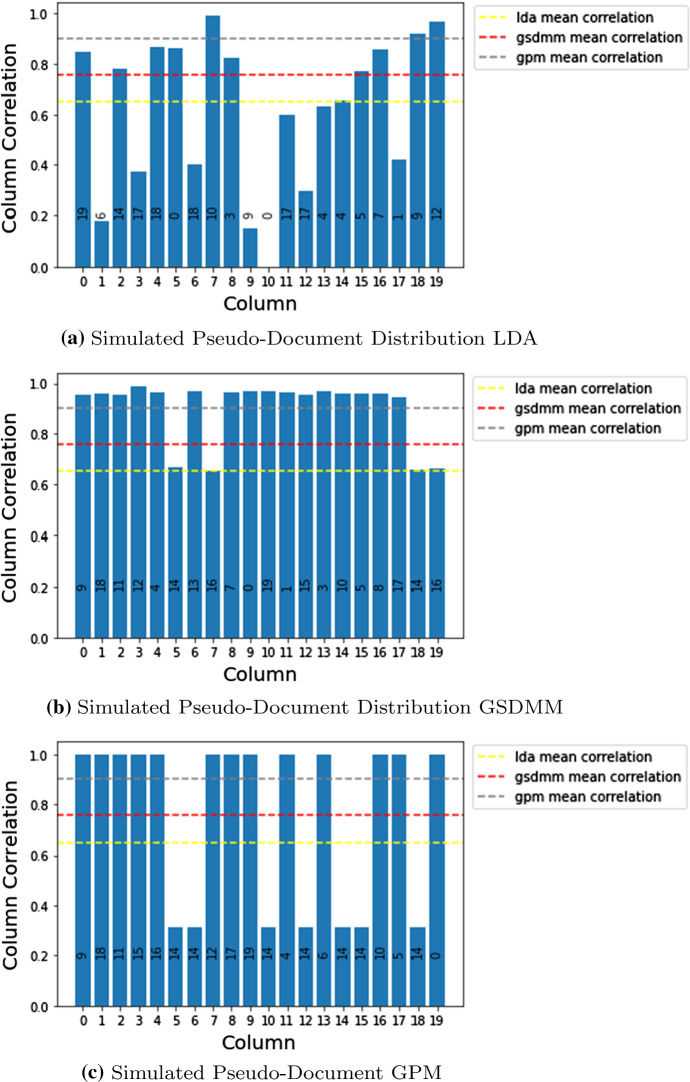
The correlation of the columns of the simulated Pseudo-Documents for $\hbox {K} = 20$, for the LDA, GSDMM, and GPM, and the theoretical $\varvec{\theta }$ matrix

**Fig. 9:**
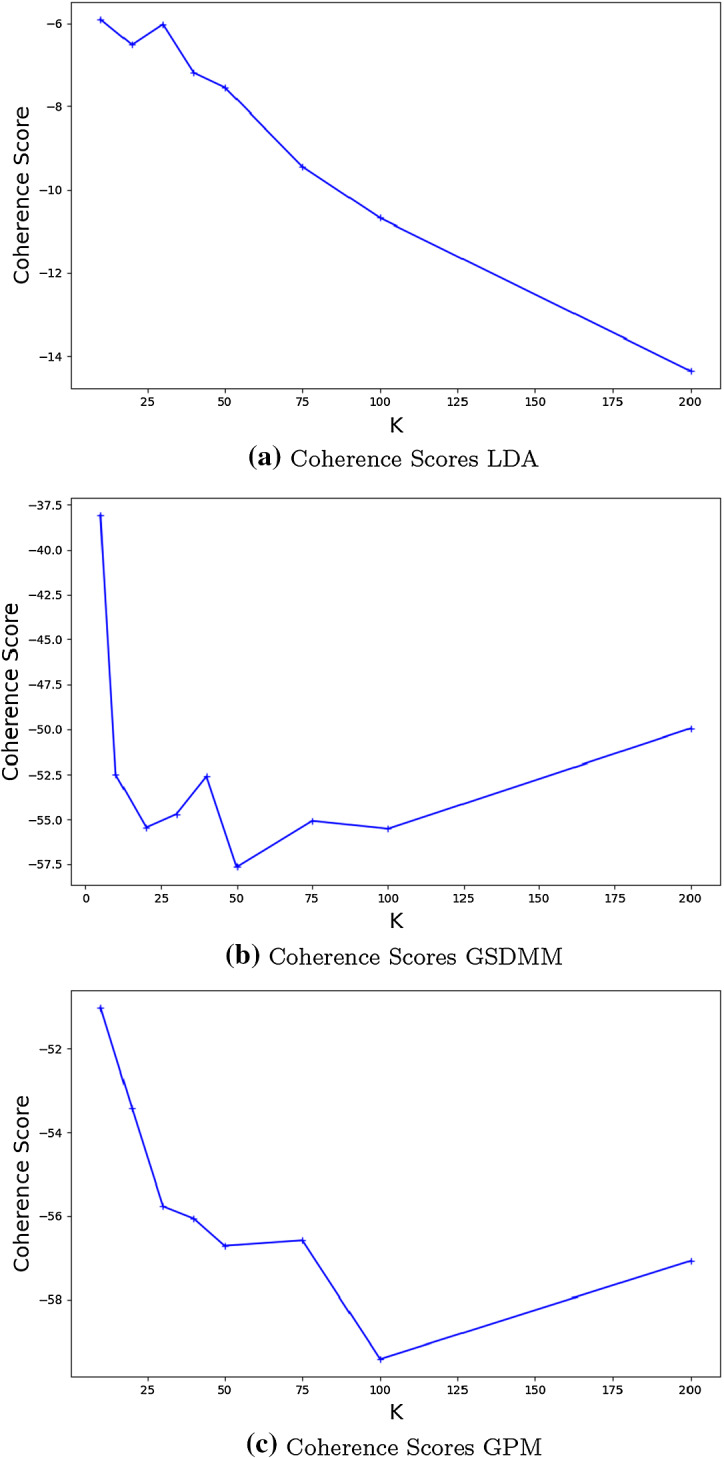
Coherence scores for different K, for the LDA, GSDMM and GPM

In addition, we compute the column correlations of the $\varvec{\theta }_{S}$ matrix with the theoretical $\varvec{\theta _{T}}$ matrix. For a perfect model fit, we would expect a unique column-wise mapping and column correlations of 1 and 0. Figure 8 shows that the LDA has the lowest column correlation, while the GPM has the highest column correlation. The column correlation of the GSDMM is lower than the GPM column correlation, but higher than the LDA column correlation. This implies that the GPM performs better than the GSDMM while GSDMM performs better than the LDA.

## Conclusion

We simulate pseudo-documents and use standard topic model evaluation methods to compare the performance of the LDA topic model with the GSDMM and GPM models for sparse and short text. In a case study, we use tweets filtered by keywords relating to the Covid-19 pandemic. The coherence scores suggest that the LDA clearly outperforms both the GPM and the GSDMM model, although GPM and GSDMM are expected to perform better, since there are specifically designed for sparse and small text data. In contrast, the model comparison with the Pseudo-Document Simulation method shows that the LDA model is outperformed by the GSDMM and the GPM models. The Pseudo-Document Simulation enables researchers to compare topic models, but our proposed solution still requires the separate hyperparameter optimization for the individual models with coherence scores or the use of priors that are recommended in the literature for the specific models. In further research, the Pseudo-document simulation approach could be also used to optimize hyperparameters of individual models with respect to the average column correlations of the $\varvec{\theta }_{S}$ matrix and $\varvec{\theta _{T}}$ matrix.

## Figures and Tables

**Fig. 3:**
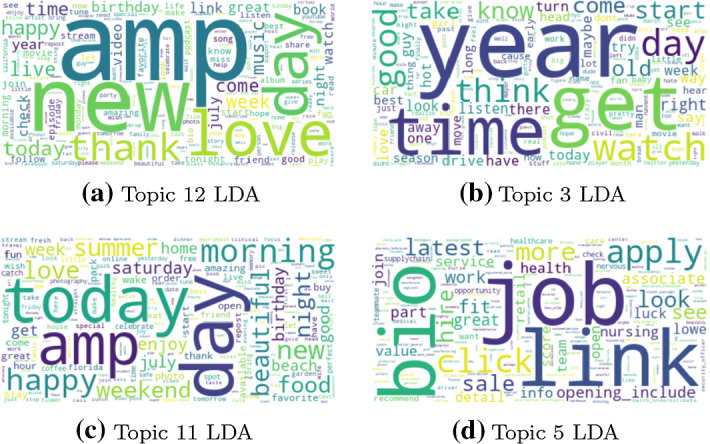
Wordclouds for the most relevant topics for the LDA

**Fig. 4:**
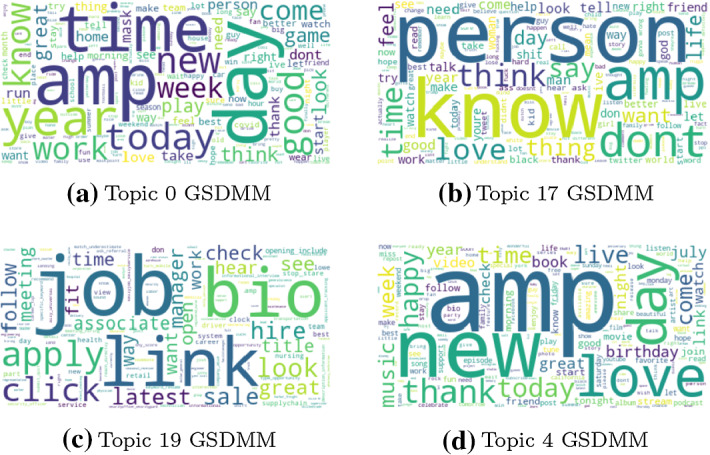
Wordclouds for the most relevant topics for the GSDMM

**Fig. 5:**
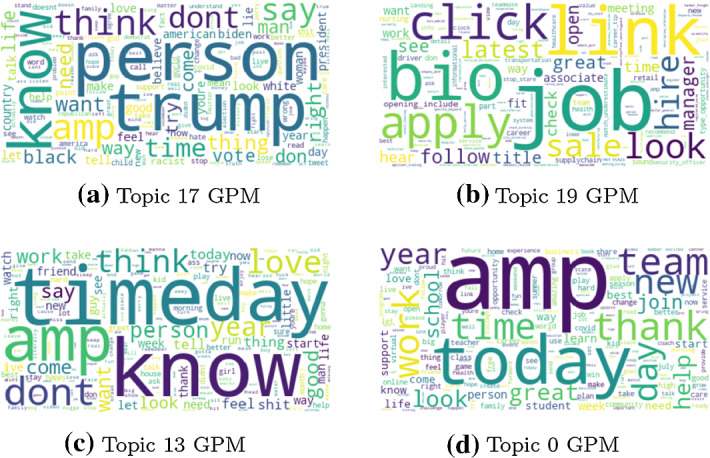
Wordclouds for the most relevant topics for the GPM

## Appendix A: Top words and Wordclouds

See the Tables 3, 4, 5 and Figs. 2, 3, 4, 5.

**Table 3:** Top words of the topics of the LDA

Topic	Top words	Given topic name
0	Job follow hear title time career view sound new post	“Job”
1	Person Don know say mask think amp covid want thing	–
2	New music art video York song beat future serve piece	–
3	Year get time watch day good think start know take	–
4	California water news fact dog local shot citizen later folk	–
5	Job link bio apply click more latest look sale hire mile	“Job market”
6	Nice police kellyjob_kellyservice hell nature kelly_service eye sun island bar	–
7	Pass speak second interview episode rep term pick worker benefit	–
8	Amp sign picture company thank help Chicago design please much	–
9	Street federal ice_cream drink series Jesus fly attack fine list	–
10	Church other ride heat self important isn’t respect comment health	–
11	Day today amp morning happy new summer love beautiful weekend	–
12	Amp thank love life work god great family day time	–
13	Trump black white vote woman president American man power amp	“Politics”
14	Sunday run play game win lake Monday last time red	–
15	Great way interested check meeting Don security ask_referral shy_score station	–
16	Use want right system look land blue manager drop keyword_resume	–
17	Don ready check know person reach_directly company_able submit_quit color look	–
18	School group team high teacher learn education class student join	“School”
19	Fall racist ive gonna course bit heart goal donate sorry	–

**Table 4:** Top words of the topics of the GSDMM

Topic	Top words	Given topic name
0	Day get amp time year today good know work week	–
1	Steve look here case last July angel thealienist nerve help	–
2	New love amp available hair look mask fashion color shop	“Fashion”
3	Game amp team mile win foot_speed today mph_squawking play run	“Sports”
4	Amp new love day thank today happy live time come	–
5	Amp food today good chicken day new dinner get love	“Food”
6	Amp new king report Mississippi country hes murder include police	“Crime”
7	CDT station edt report_tstm mesonet_report	“Weather”
	Tree report heavy_rain wnd_dmg inch_mesonet	
8	Art new photography amp York artist love episode painting draw	“Art”
9	Amp today new open beer day order weekend Saturday available	“Weekend”
10	Exit beach day park beautiful morning new summer love lake	“Summer”
11	John_Lewis right rest amp thank goodtrouble johnlewis civil life good	“John Lewis”
12	Team amp join today work school thank help new look	–
13	Music new hiphop rap rapper link amp newmusic now artist	“Music”
14	Amp happy make again quote lack modern have part style	–
15	Fitness workout amp morning day run today good motivation let	“Fitness”
16	Trump person amp covid mask know say right think don’t	“Trump and covid”
17	Person know amp don’t think time love get say thing	–
18	Amp help please thank Jamesnewheart much America	“Donate”
	Donate transplant$_p$lease care	
19	Job link bio apply click look sale hire more latest	“Job offers”

**Table 5:** Top words of the topics of the GPM

Topic	Top words	Given topic name
0	Amp today thank team work time day year great new	–
1	New home amp today love thank get come realestate look	“Housing”
2	Day happy love new birthday today fitness morning workout get	“Fitness”
3	Mile foot_speed mph_squawking Los_Angeles Eurocopter_circle	–
	California circle new art school	
4	Amp love time watch year game day get play know	–
5	CDT station mesonet_report edt heavy_rain inch_mesonet	“Weather”
	Report_tstm tree rain_inch report_heavy	
6	Day new amp park morning lake sunset beautiful beach today	“Summer”
7	Get game year amp home love play day time good	–
8	New amp live link get episode check stream today thank	“Entertainment”
9	Amp new today love day happy available thank open look	–
10	Amp day today new food get love good time summer	–
11	New amp love music beach Florida day today California summer	“Summer”
12	Exit RTE construction street station direction avenue	“Traffic”
	Line update_incident road	
13	Get time know day amp don’t love think year person	–
14	John_Lewis right rest thank goodtrouble John Lewis amp life good god	“John Lewis”
15	Amp new day today time love get come night thank	–
16	Mask person covid amp wear need know trump school don’t	“Covid-19”
17	Person trump know amp think say don’t time get thing	“Trump”
18	Amp help please thank support America much donate	“Donation”
	Care Jamesnewheart	
19	Job link bio click apply look more sale hire latest	“Job offers”

## Appendix B: Data and pseudo-document simulation

See the Figs. 6, 7 and 8.

## Appendix C: Online variational Bayes for LDA

The following algorithm assumes symmetric Dirichlet priors such that $\varvec{\lambda }_{\alpha } = ({\lambda }_{\alpha },\ldots ,\lambda _{\alpha })$ and $\varvec{\lambda }_{\beta } = ({\lambda }_{\beta },\ldots ,\lambda _{\beta })$. We also have that $q(z_{di} = k) = \psi _{dw_{di}k}$, where $\psi _{w_{di}dk}$ are the components of $\varvec{\psi }_{w_{di}d}$. Additionally, the expectation of $\log \varvec{\theta }$ and $\log \varvec{\beta }$ with respect to *q* are analytically accessible via the gamma function $\Psi$: ${\mathbb {E}}_q[\log \theta _{dk}] = \Psi (\kappa _{dk}) - \Psi (\sum _{i=1}^K \kappa _{di})$ and ${\mathbb {E}}_q[\log \beta _{kw}] = \Psi (\gamma _{kw}) - \Psi (\sum _{i=1}^K \gamma _{ki})$. Now, the algorithm for online Variational Inference for LDA, introduced by Hoffman et al. (2010), takes the following form:

## Appendix E: Coherence scores

See the Fig. 9.

## Footnotes

1 Note that Mazarura et al. (2020) created the GPyM_TM package which implements both the GPM as well as the GSDMM model in Python and is used for estimations in this work.

2 e.g. *“Bayesian statistics" and “World War 2"*.
